# Evidence-Based Evaluation of eHealth Interventions: Systematic Literature Review

**DOI:** 10.2196/10971

**Published:** 2018-11-23

**Authors:** Amia Enam, Johanna Torres-Bonilla, Henrik Eriksson

**Affiliations:** 1 Centre for Healthcare Improvement Technology Management and Economics Chalmers University of Technology Gothenburg Sweden

**Keywords:** evidence-based practice, program evaluation, systematic review, technology assessment

## Abstract

**Background:**

Until now, the use of technology in health care was driven mostly by the assumptions about the benefits of electronic health (eHealth) rather than its evidence. It is noticeable that the magnitude of evidence of effectiveness and efficiency of eHealth is not proportionate to the number of interventions that are regularly conducted. Reliable evidence generated through comprehensive evaluation of eHealth interventions may accelerate the growth of eHealth for long-term successful implementation and help to experience eHealth benefits in an enhanced way.

**Objective:**

This study aimed to understand how the evidence of effectiveness and efficiency of eHealth can be generated through evaluation. Hence, we aim to discern (1) how evaluation is conducted in distinct eHealth intervention phases, (2) the aspects of effectiveness and efficiency that are typically evaluated during eHealth interventions, and (3) how eHealth interventions are evaluated in practice.

**Methods:**

A systematic literature review was conducted to explore the evaluation methods for eHealth interventions. Preferred reporting items for systematic reviews and meta-analyses (PRISMA) guidelines were followed. We searched Google Scholar and Scopus for the published papers that addressed the evaluation of eHealth or described an eHealth intervention study. A qualitative analysis of the selected papers was conducted in several steps.

**Results:**

We intended to see how the process of evaluation unfolds in distinct phases of an eHealth intervention. We revealed that in practice and in several conceptual papers, evaluation is performed at the end of the intervention. There are some studies that discuss the importance of conducting evaluation throughout the intervention; however, in practice, we found no case study that followed this. For our second research question, we discovered aspects of efficiency and effectiveness that are proposed to be assessed during interventions. The aspects that were recurrent in the conceptual papers include clinical, human and social, organizational, technological, cost, ethical and legal, and transferability. However, the case studies reviewed only evaluate the clinical and human and social aspects. At the end of the paper, we discussed a novel approach to look into the evaluation. Our intention was to stir up a discussion around this approach with the hope that it might be able to gather evidence in a comprehensive and credible way.

**Conclusions:**

The importance of evidence in eHealth has not been discussed as rigorously as have the diverse evaluation approaches and evaluation frameworks. Further research directed toward evidence-based evaluation can not only improve the quality of intervention studies but also facilitate successful long-term implementation of eHealth in general. We conclude that the development of more robust and comprehensive evaluation of eHealth studies or an improved validation of evaluation methods could ease the transferability of results among similar studies. Thus, the resources can be used for supplementary research in eHealth.

## Introduction

### Background

The use of electronic health (eHealth) is still driven by assumptions about the benefits of eHealth rather than its evidence [1]. With time, the trustworthiness and robustness of eHealth to facilitate safe and cost-efficient care are being questioned because of a lack of evidence [2]. This may trigger reluctance in investing and developing policies related to eHealth in organizations as well as in countries [3].

The term *eHealth* was introduced in the 1990s [4]; however, it was hardly in use until 1999 [5]. According to Eysenbach [5]:

e-health is an emerging field in the intersection of medical informatics, public health and business, referring to health services and information delivered or enhanced through the Internet and related technologies.

Eysenbach believes eHealth stands for more than internet and medicine [5]. In our study, eHealth was used as the broadest umbrella encompassing everything that comes within information and communication technology and health care, including telemedicine, mobile health, and health informatics.

Systematic evaluation can capture the evidence and criteria that evaluative judgment is based on and curtail the sources of biases [6]. The quality of an evaluation is assessed by the credibility of evidence assembled through it and using evidence in refining the policies and programs [7]. Evaluation of eHealth interventions is complex because of several reasons (eg, the need for multidisciplinary collaboration [8], context dependency [9], and differences in epistemological beliefs considering the interventions in clinical studies or including social aspects as well [10-14]). Therefore, variety exists concerning how the evaluation of eHealth interventions is performed and presented. Garnering robust evidence through evaluation becomes difficult because of these circumstances.

It is relevant to understand evidence-based medicine (EBM) while discussing the importance of evidence in eHealth interventions. A common query is how EBM can help generate evidence for eHealth interventions [15]:

Evidence-based medicine is the conscientious, explicit, and judicious use of current best evidence in making decisions about the care of individual patients. The practice of EBM means integrating individual clinical expertise with the best available external clinical evidence from systematic research.

As per this definition, *evidence* in EBM is conspicuously related to the clinical aspect. Although it is argued whether EBM is only about randomized controlled trials [16] or not [15], it is quite explicit that EBM usually does not contemplate anything outside clinical practices. However, eHealth interventions have more aspects to evaluate besides the clinical aspects. An extensive assessment of the aspects including sociotechnical aspects is needed through each phase of the technology’s life cycle while evaluating eHealth interventions [10,11,17]. Hence, to gather evidence from eHealth intervention, the evaluation process requires a distinct approach than what is usually put forward within EBM.

### Objective

Our objective was to elucidate how the evidence of effectiveness and efficiency of eHealth can be generated through evaluation. Consequently, a literature review was conducted to understand the evaluation process regarding both theories of eHealth and the practices in case studies of eHealth interventions. We decided to employ a broader perspective at the beginning of the review process to achieve our research objective. As the literature review progressed, our research objective narrowed, and the research questions were redefined several times. However, the objective was always to understand the evaluation of eHealth from a comprehensive perspective. It was pertinent to recognize the phases of eHealth interventions where evaluation occurs and the aspects of effectiveness and efficiency that are evaluated during such interventions. Our 3 research questions were as follows:

1. How is evaluation conducted in distinct eHealth intervention phases?
2. What aspects of effectiveness and efficiency are typically evaluated during eHealth interventions?
3. How have eHealth intervention case studies been evaluated?

Finally, we presented an approach to evaluate eHealth interventions by developing a model—Evidence in eHealth Evaluation. To our knowledge, this model is a novel way of looking into evaluation of eHealth interventions for comprehensive evidence. This conceptual model was based on the findings of the literature review.

## Methods

### Systematic Review

#### Identification and Screening

A systematic search of relevant literature was conducted following preferred reporting items for systematic reviews and meta-analyses (PRISMA) guidelines [18]. Google Scholar and Scopus were used to search the following identified terms, “research methods” and “eHealth interventions,” “study design” and eHealth interventions,” “evaluation methods” and “eHealth interventions,” “eHealth interventions” and “evaluation framework,” “evidence based” and “evaluation,” and “eHealth interventions.” The selected set of terms is aligned to the broad scope of eHealth interventions’ evaluation methods and to the aim of including and analyzing as many relevant studies as possible in the review. We included scientific papers published between 1990 and 2016. As the term eHealth evolved during the 1990s [4], we deemed it reasonable to consider the literature published on eHealth interventions since then. A total of 1624 records were found with these selected search keywords.

The screening of the papers was conducted in 3 steps. For the first 2 steps, the screening was based on the title of the manuscripts using a predefined set of exclusion and inclusion criteria. Only scientific papers were used, whereas books and patents were excluded during the search. To avoid overanalysis and repetition of the papers, the exclusion criteria for the first step were citations, literature reviews, and meta-analyses. In addition, studies addressing specific health issues designed to answer clinical research questions were excluded (ie, publications solely addressing behavior change theory, ergonomics, drugs, sedentary issues, or physical activity intervention as well as those addressing nonadult patients). The number of records was reduced to 813 after the first elimination. At this point, all the records were listed together, and duplicate records were removed. During the second step, title screening was conducted. For papers whose titles did not explicitly mention the intervention target group, the abstracts were read to decide. When in doubt, the papers were included for further scrutiny in the next step. Consequently, only those records that included either a conceptual discussion about eHealth interventions or discussion about eHealth interventions that focused on adult patients and caregivers were selected. The third step of the screening process started with 279 records; this time, all the abstracts and the methodology of the papers were read by 2 of the authors individually. Previously, we devised the inclusion parameters for this stage; specifically, the selected papers ought to (1) be aligned to the study objective, (2) have the potential to provide insight for 1 or more research question, and (3) comply with the inclusion and exclusion criteria described above. At the end of step 3, the authors discussed their observations, and 81 papers were selected for thorough reading and analysis. Besides the papers selected through systematic search, 10 records were added for further analysis. These records were found from the citation of the papers selected using the systematic literature search. All 10 papers were included in this study because of their relevance to our objective.

#### Eligibility and Inclusion

**Figure 1:**
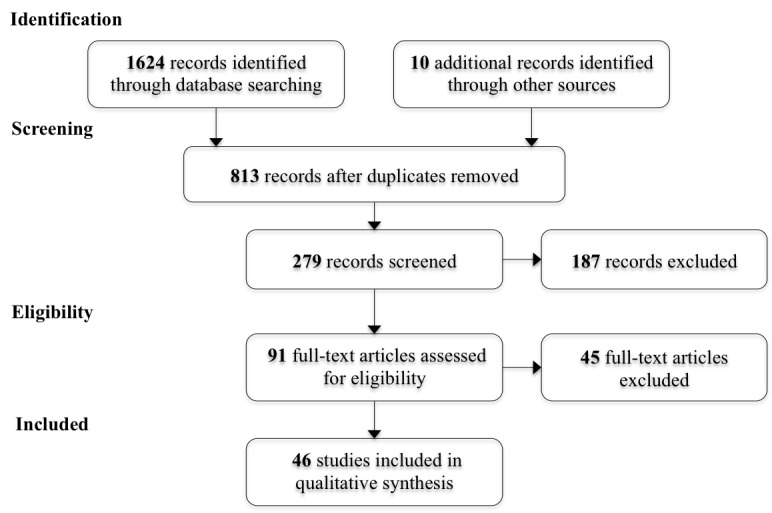
Preferred reporting items for systematic reviews and meta-analyses flow diagram of the study selection process.

To extract and record useful information from the papers and to gain a general overview of evaluation in eHealth interventions, a Microsoft Excel spreadsheet was created with the criteria shown in Multimedia Appendix 1. Although most of the criteria were adopted from section 2.2.3 *Planning the topic and scope of a review* in the Cochrane review [19], criterion such as *learning point* was included by us. This section of Cochrane review was adapted to identify the potentials of the selected papers in fulfilling the research objectives. On the other hand, it seemed advantageous to record novelties of the studies concerning *learning points* for our own development. During these phases, the papers (N=91) were read meticulously, which led to the final screening. As a result, 46 papers were selected for the qualitative analysis that categorically focused on evaluation in terms of phases and aspects. The flow diagram of the papers selection process is presented in Figure 1.

### Qualitative Analysis

On the basis of the summary of studies mentioned in the previous section, the papers (N=46) were classified into 2 categories: (1) conceptual exploration of eHealth interventions (n=21) and (2) case studies of eHealth interventions (n=25). Using the summary table (Multimedia Appendix 1), the papers in the first category were divided into 2 groups: (A) evaluation of distinct eHealth intervention phases (n=10) and (B) aspects of evaluation in eHealth interventions (n=11).

#### Evaluation of Distinct Phases of an Electronic Health Intervention

For the papers in group A, thematic scrutiny was applied by mapping out the content of the papers and grouping the phases of intervention with similar objectives, activities, or results. The objective of the analysis was to understand whether the researchers emphasize some phases over others during evaluation and, if so, what phases are most frequently evaluated during an intervention.

#### Aspects of Electronic Health Intervention Supposed to Be Evaluated According to Electronic Health Literature

The studies in group B elaborated on the aspects evaluated to gather evidence of the efficiency and effectiveness of eHealth evaluations. These aspects and their key area of measurements were extracted from the papers to understand the parameters of efficiency and effectiveness that are emphasized by eHealth literature.

#### Evaluation Reported in Empirical Studies of Electronic Health Interventions

The studies categorized as case studies of eHealth interventions were analyzed based on several characteristics (ie, duration of the intervention, number of participants, used a framework or predefined theory for evaluation or designing the intervention, aspects assessed for evaluation, phases involved in the evaluation, data collection method, and presentation of intervention results). The purpose of the analysis was to conduct a descriptive comparison of the characteristics of evaluation performed in case studies with that of the conceptual papers.

## Results

### Evaluation of Distinct Phases of an Electronic Health Intervention

This subsection concentrates on how evaluation is conducted in distinct phases of an eHealth intervention. From the selected studies in group A, a spectrum of phases of an eHealth intervention was identified including *design, pretesting, pilot study, pragmatic trial, evaluation*, and *postintervention*. Table 1 provides a compilation of the phases along with the area of focus and key activities within each phase.

It can be ascertained from Table 1 that evaluation is not commonly performed in the design and pretesting phases. Although some researchers see evaluation as an ongoing process throughout the intervention [12,21,23], others believe there is value in evaluating the intervention at the end of the study period [12]. Concerning the latter, evaluation itself is one of the phases of the intervention.

**Table 1:** Characteristics of distinct phases of an electronic health intervention.

Phase	Area of focus	Key activities
Design phase	Conceptualization	Gather theoretical foundations and empirical evidence to detect the existing problems and identify viable solutions [20-24] and define the objectives of the to-be-developed technology [20,25]
	Contextual inquiry	Identify the end users and stakeholders to define and analyze the characteristics of the context the technology is going to be implemented on [20,21,23-25]
	Value specification	Prioritize the critical values of the technology derived from the end users and stakeholders’ needs [12,22-25]
	Requirements specification	Translate the values into functional and technical requirements that frame the final design and the technology development [12,23-25]
Pretesting phase	Conduct short-term trials	Provide evidence of efficacy of the technology [12,24,26]; measure factors such as optimal intensity, timing, safety, feasibility, usability, intervention content, and logistic issues [21,22,26]; and evaluate the correspondence between technology capabilities and technology requirements [24]
Pilot study	Strategic plan	Define the preliminary plan of the pilot study (ie, objective, timeline, budget, sponsors, and team members [27,28] and identify related ethical and legal issues [20,28]
	Study design	Define the study type, duration, and participants [20,26,28] as well as data collection methods [28] and design the recruitment process to conform to statistical validity and minimize selection bias [27]
	Evaluation	Evaluate the technology and its impact simultaneously [27] and evaluate the effectiveness of the intervention [22]
Pragmatic trial phase	Execution	Administer the intervention to a larger group of participants [21,23,26] with fewer eligibility restrictions [26]
	Evaluation	Formative and summative evaluation (discussed in the evaluation phase) and internal and external evaluation (discussed in the evaluation phase)
Evaluation phase	Formative evaluation	Generate measures that provide timely feedback [12,23] and perform an evaluative iterative process, as the findings from each step are used to inform subsequent steps [21]
	Summative evaluation	Provide generalizable knowledge and benefits of the intervention [12,23]
	Internal evaluation	Perform an evaluative process intrinsic to information and communication technology implementations and conducted by the implementation team [12]
	External evaluation	Conduct the evaluation by external evaluators to provide expertise where it is needed and minimize the bias of in-house evaluators [12]
Postintervention phase		Conduct postmarketing or surveillance studies to follow up the technology once scaled up and used by a wider audience [22,26]

### Aspects of Electronic Health Intervention Supposed to Be Evaluated According to Electronic Health Literature

We determined the aspects that researchers evaluated to gather the evidence of efficiency and effectiveness of an eHealth intervention. To understand the aspects of efficiency and effectiveness, we compiled the dimensions of eHealth interventions that are proposed to be measured by the studies categorized in group B (n=11). While excerpting the dimensions during the qualitative analysis, we found that they can be classified into 7 aspects: *organizational aspect*, *technological aspect*, *human and social aspect*, *clinical aspect*, *cost and economic aspect*, *ethical and legal aspect*, and *transferability aspect*. Table 2 exhibits these aspects along with their key area of measurements.

**Table 2:** Description of identified aspects of evaluation in electronic health interventions.

Aspects of assessment	Key areas of measurement
Organizational aspect	Organizational setting where the intervention is taking place; it can differ depending on the scale of the intervention (eg, health center, region, and country) [29]; all type of individuals or groups in the health care system that participate in the eHealth intervention, their characteristics, and expectations [30]; organizational performance and professional practice standards [30]; changes in the functions of the health care provider, skills and resource demands, and the roles of the professionals in the organization [29,31-33]; representativeness and participation rates of the health care professionals during the intervention [34]; capability of the organization to implement the intervention [30,34-38] and the extent that the technology fits the organizational strategy, operations, culture, and processes [30]; and sustainability or the degree that the technology becomes accustomed in the daily practice of an organization [29,32,34]
Technological aspect	Ensure trust [38], effectiveness, and contribution of quality of care [30,36] of the technology implemented; *system performance*: hardware and software requirements, correct functioning of the components [29,38], and system capability to meet users’ needs and fit the work patterns of the health care system’ professionals [30,39,40]; *usability*: broad experience of the users with the system [29,33,37,40]; *privacy and security:* safety and reliability of the technology [29], and security of the data managed in the technology [37,38]; *technical accuracy:* quality of the transfer of data [41]; *information quality*: relates to accuracy, completeness, and availability of the information produced by the system (eg, patients’ records, reports, images, and prescriptions), and it depends on users’ subjectivity [30,39]; *service quality:* measures the support and follow-up service delivered by the technology provider [39]; *triability:* the ability of the innovation to be tested on a small scale before the final implementation [40]; *maturity:* whether the system has been used on a sufficient number of patients to address all the technical problems [36]; and *interoperability*: communication between the technology and the pre-existing systems, the fit between the technology and the existing work practices [37]
Human and social aspect	Acceptance and usability satisfaction of the technology used in the intervention [30,31,33,36,38,39,41] where the user can be physicians, nurses and other staff, and patients, depending on the type of the participants in the intervention [41]; *system use:* volume of use, who is using, purpose of use, and motivation to use the technology [39]; *user satisfaction:* perceived usefulness, enjoyment, decision-making satisfaction, and overall satisfaction for the technology [30,39]; and psychological aspects such as satisfaction, well-being, and other psychological variables, and social aspects such as accessibility to the technology, the social relationships evolving over the transmission of care, or activities of the patients under the intervention [31]
Clinical aspect	Benefits and unanticipated negative effects of the intervention, biological outcomes including disease risk factors, behavioral outcomes of the participants, staff who deliver the intervention and the sponsors, and quality-of-life outcomes to evaluate participants’ mental health and satisfaction [34] and long-term measurements of the diagnostic and clinical effectiveness [41,35,36], safety of care [33,35,36], and quality of care [33]
Cost and economic aspect	Cost analysis methods to compare the intervention with relevant alternatives in terms of costs and consequences [36]; diverse cost analysis methods can be considered (eg, cost-minimization analysis, cost-effectiveness analysis, cost-benefit analysis, cost-utility analysis, and cost-consequence analysis) [30,31,41] and are conducted from several perspectives such as societal, third-party payers, health care providers, or patient [31]; and diverse costs can be included such as investment cost, monthly user charge of equipment, costs of used communication line, education of the technology, costs of patients and their close relatives [41], wages of doctor and other staff [30,41], expenditure and revenue for the health care organization adopting the technology [36], and resource utilization and opportunity cost of the eHealth intervention [34]
Ethical and legal aspect	Ethical concerns of the app itself and its implementation including all the stakeholders’ viewpoints on using the technology and the key ethical principles associated with the context in which intervention is conducted [35,36] and legal aspect identifies and analyzes the legislative documents and legal obligations that may exist in each context involved in the intervention [30,35,36]
Transferability aspect	Participation and representativeness of the intervention, percentage of persons who receive or are affected by the program, and the characteristics of participants and nonparticipants to investigate the extent that participants are representative and what population group should be a priority for future research [34] and transferability of results from studies of eHealth from one setting to another and the assessment of validity and reliability of the study [36]

### Evaluation Reported in Case Studies of Electronic Health Interventions

The papers categorized as case studies of eHealth intervention show substantial variation in the approaches taken to evaluate the interventions. The use of standardized frameworks and theories for evaluating the interventions was hardly noticed in these studies. Multimedia Appendix 2 provides the result of the analysis [20,42-65].

To summarize Multimedia Appendix 2, it can be said that out of 25 case studies, 16 (64%, 16/25) evaluate clinical aspects, 12 (48%, 12/25) evaluate human and social aspects, 5 (20%, 5/25) evaluate technological aspect, and 4 (16%, 4/25) evaluate organizational aspect. The other aspects discussed by the theory-based literature (Table 2) are not evaluated in any of the case studies.

## Discussion

### Principal Findings

From the papers reviewed in this study, it has been revealed that numerous approaches to conceptualize and conduct eHealth intervention coexist. Several attributes of evaluation of eHealth intervention have become known through this review. There are vivid differences between how evaluation is conducted in practice (case studies) and how it is discussed in the conceptual papers. Moreover, a wide range of variety prevails within each group. Evaluation has been depicted as both static action performed at the end of the intervention [20,24,26-28] and dynamic action dividing it further into summative and formative evaluation [12,21,23]. Depending on the evaluators, evaluation can also be classified into internal and external assessment [12]. However, all case studies conducted evaluation at the end of the intervention. Although several aspects of evaluation have been found in conceptual papers [32-39,41], the case studies mostly evaluated clinical [20,42-44,46-49,52,53,55,59-61,64,65] and human and social aspects [42,46-49,53-55,59,61,63,65].

Although analyzing standardization of eHealth evaluation was not an objective of this review, the variability found in the studies compelled us to think whether it hinders the sharing of evidence among eHealth interventions. Scarcity of evidence, in turn, could delay the growth of eHealth. It is noticeable that the need for evidence is not clearly stated in any of the papers. The evaluation of the empirical studies typically focused on the success or failure of the technology (eHealth) in that intervention. It seems that the numerous efforts taken in eHealth research are still quite disconnected, and they are thus unable to create a synergic effect on the growth of eHealth.

### Evidence in Electronic Health Evaluation Model

**Figure 2:**
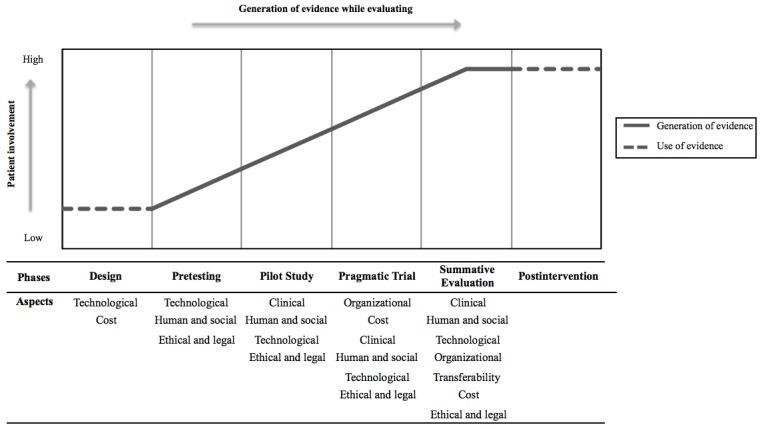
The evidence in electronic health (eHealth) evaluation model.

It was noticeable from the review that although some studies elaborate on the aspects of evaluation for eHealth intervention (studies from group B) and some organize the evaluation of intervention into certain phases (studies from group A), no visible interaction has been made so far between these 2 groups of works (ie, what to assess during what phase). There is a gap where a connection can be made between the distinct phases of intervention and aspects of evaluation. This led us to develop the Evidence in eHealth Evaluation model (Figure 2), which exhibits the accumulation of evidence by assessing certain aspects of evaluation in distinct intervention phases. The Evidence in eHealth Evaluation model is a novel approach to investigate the evaluation of eHealth interventions.

In this study, an eHealth intervention comprising all 6 phases (ie, *design, pretesting, pilot study, pragmatic trial, evaluation,* and *postintervention*) was conceived as a comprehensive intervention. We propose that the generation of robust evidence of effectiveness and efficiency would be plausible when the evaluation is conducted through all intervention phases. Moreover, the aspects of evaluation (ie, *organizational aspect, technological aspect, human and social aspect, clinical aspect, cost aspect, ethical and legal aspect,* and *transferability aspect*) would vary in each phase depending on activities of the phases. For example, when an eHealth intervention initiates with the *design* phase, the decisions are made based on the evaluation of the *technological* aspect and *cost* of technology development. The formal evaluation of the intervention begins in succeeding phases. The evaluation of *technological*, *human and social*, and *cost* aspects occurs in the *pretesting* phase. During the *pilot study* phase, the focus of evaluation shifts primarily to *clinical* aspect followed by *human and social*, *technological*, and *ethical and legal.* Depending on the evidence garnered in the pilot study, the intervention may proceed to the next phase or go back to the *design* phase. As the intervention is scaled up in the *pragmatic trial*, the evaluation is conducted to identify whether the technology-enabled care can be executed within the realistic layout of an organization. Hence, the key areas of evaluation in this phase are *organizational* and *cost* aspects along with other aspects such as *clinical, human and social, technological*, and *ethical and legal*. The last phase of gathering evidence is *summative evaluation*, where all the aspects are assessed including transferability. This comprehensive evaluation process gradually accumulates the evidence that reaches its peak in the *summative evaluation* phase and is used in the *postintervention* phase to make future decisions. The model also exhibits how the involvement of patients increases continuously from the *design* phase to the *pragmatic trial*, escalating the complexity of the evaluation process.

The inclusion of relevant information regarding other aspects besides the clinical aspect (eg, organizational aspect and cost aspect) allows creating reusable knowledge to facilitate the transfer of results to other settings [36] and to obtain useful insights for long-term implementations. It can be assumed that assessing all the aspects in a single study might conclude with a confounding result, as all the aspects are interrelated and inferior performance in an aspect can affect the performance in other aspects, which might create a misleading result. Therefore, our model proposes to extend the evaluation process throughout the 6 phases of eHealth intervention. The underlying idea is to assess specific aspects in each phase instead of evaluating all aspects in a single phase. This way of evaluating eHealth interventions can capture comprehensive evidence that is usually dynamic and complex in nature.

We acknowledge the fact that an eHealth intervention including all the phases presented in the model will become cumbersome because of high resource consumption. This conceptual model is not a prescription but just a way to show the progression of evidence in eHealth intervention in a reliable manner.

### Conclusions

To date, the importance of evidence has not been discussed as rigorously as the diverse research approaches and evaluation frameworks have been discussed. In this study, the Evidence in eHealth Evaluation model was developed to exhibit how evidence can be generated by evaluating certain aspects in each intervention phase. Assessing distinct aspects during distinct phases is a novel concept discussed in this study and requires further analysis. Moreover, this study implies an inconsistency between the literary concepts and practices of eHealth intervention, which has not been noted until now.

As health interventions are context-specific, the transferability of results from eHealth studies may be difficult. Moreover, neither the conceptual nor the case studies suggested the long-term implementation of specific technology into the health care settings where it has been tested. We believe that this might be caused by a lack of or insufficiency of preliminary evidence of the effectiveness and efficiency after conducting the micro-trials or short-term tests on the effects of the technology. Consequently, it appears that lack of evidence hinders the growth of eHealth. Further research directed toward evidence-based evaluation can not only improve the quality of that intervention study but also facilitate long-term implementation of eHealth in general. We conclude that the development of more robust and comprehensive evaluation of eHealth studies or an improved validation of evaluation methods could ease the transferability of results among similar studies. Thus, the resources can be used for supplementary research in eHealth.

### Limitations

This study is not devoid of limitations. We tried to include and analyze as many papers as possible; however, unknowingly and unintentionally, some papers may have been omitted. Furthermore, regarding the model, its development is in the preliminary stages; therefore, it cannot be compared with other validated frameworks.

## Appendix

Multimedia Appendix 1 Criteria of the summary table developed to record information from the selected papers.

Multimedia Appendix 2 Evaluation reported in empirical studies of eHealth interventions.

## Abbreviations

EBM: evidence-based medicine
eHealth: electronic health
PRISMA: Preferred reporting items for systematic reviews and meta-analyses
