# Supplementary material for: Evidence-Based Evaluation of eHealth Interventions: Systematic Literature Review
Source: J Med Internet Res. 2018 Nov 23;20(11):e10971. doi: 10.2196/10971 (PMC6286426; doi:10.2196/10971)
Supplement: Multimedia Appendix 1 [file jmir_v20i11e10971_app1.pdf]

Multimedia Appendix 1. Criteria for summarizing the articles found in the systematic literature review.

**Summarizing criteria**	
**1. Objective**	In this section, the basic idea of the article is discussed focusing on the aim of the research. The main issues discussed in the article are noted here in a concise manner.
**2. Method**	With an aim to summarize the research methodology of the article, method section is categorized in following sections.
**2.1. Types of studies**	In this section, we noted whether the article is entirely conceptual, i.e. has not been connected to any empirical study of intervention, or based on any eHealth intervention.
**2.2. Background of the authors**	Whether the authors of the article are healthcare practitioners, researchers, or a combination of both.
**2.3. Types of participants**	This section records the types of participants taken part in intervention, i.e. patient, informal caregiver, formal caregiver, healthcare administrator; or the types of the people involved for the research purpose in conceptual articles; i.e. experts in related field.
**2.4. Types of outcome measures**	The ultimate outcome of the article was recorded in this section in terms of framework, model, guidelines, and evidence of efficiency, discussion, learning points.
**2.5. Data collection method and data analysis**	One of the objectives of this section was to record the data collection methods either prescribed by the authors in conceptual articles or used in the interventions, or used for writing the article. Another objective was to record the analysis method that has used to analysis the quantitative and qualitative data.<br>We classified the data collection method in three segments:<br>• Clinical (Cl), i.e. data collection through medical tests;<br>• Quantitative (Qn), i.e. data collected by using questionnaires that result in numerical scores;<br>• Qualitative (Ql), i.e. data collected through interviews and observations.
**3. Learning points**	This section was used to record the unique aspect of the article. We noted the points that seemed to be new and interesting in the article while reading it.
