# Supplementary material for: Evidence-Based Evaluation of eHealth Interventions: Systematic Literature Review
Source: J Med Internet Res. 2018 Nov 23;20(11):e10971. doi: 10.2196/10971 (PMC6286426; doi:10.2196/10971)
Supplement: Multimedia Appendix 2 [file jmir_v20i11e10971_app2.pdf]

Multimedia Appendix 2. Evaluation reported in empirical studies of eHealth interventions.

Reference	Used a framework or a predefined theory for evaluation or designing the intervention	Intervention duration	Number of participants	Data collection method	Presentation of result of the intervention	Aspects for evaluation	Phases involved
[20]	No	6 weeks	28	**Qn:** Surveys (DASS-21, WSAS, MHSES). **Ql:** Telephone interviews.	Statistically and in descriptive format.	**Clinical aspect:** Stress, anxiety, depression.	Pilot study
[42]	No	1-2 weeks	17	**Ql**: Thematic analysis of text messages.	Descriptive interpretation of data.	**Clinical aspect**: Mental health symptoms; mental health coping strategies; mental health treatment and management; lifestyle behaviors. **Human and social aspect**: Social relationships and leisure activities; motivation and personal goal setting; and independent living.	Pilot study
[43]	No	6 weeks and 12 weeks	101	**Qn:** Survey with standardized questionnaire (PHQ-9).	Statistically	**Clinical aspect:** Depressive symptom severity.	Pilot study
[44]	No	6 weeks	537	**Qn:** Survey with standardized questionnaire (HADS, CES-D).	Statistically	**Clinical aspect:** Symptoms of anxiety and depression.	Pilot study
[45]	No	2 years	49	**Qn:** Survey **Ql:** Behavioral analysis, semi-structure interviews.	Descriptive interpretation of data.	**Technological aspect:** Content, layout, program design and usability testing of a web-based program.	Design and pretesting
[46]	No	12 weeks	225	**Qn:** Survey (online questionnaire). **Ql:** Clinical interview.	Statistically	**Clinical aspect:** Disorder severity rating, number of diagnosis, general psychological distress level. **Human and social aspect:** quality of life (QoL), treatment satisfaction.	Pilot study
[47]	No	6 months	38	**Qn:** Survey with	Statistically	**Clinical Aspect:** Psychiatric	Pilot study

				standardized questionnaires (BPRS, SANS, IMR, PHQ-9, SF-12).		symptoms, health indicator. **Human and social aspect:** QoL, illness self-management.	
[48]	No	73.2 days on average	2632	**Qn:** Survey with standardized questionnaire (KPD-38).	Statistically	**Clinical Aspect:** Physical and psychological impairment. **Human and social aspect:** Social problems.	Pragmatic trial
[49]	No	11 months	241	**Qn:** Survey with standardized questionnaires (PSS, COPE-index, GSE, WHO-5) and a single question for QoL).	Statistically	**Clinical aspect:** Stress level. **Human and social aspect:** caring situation, well-being, QoL, self-efficacy.	Pilot study
[50]	No	12 months	Patients from 20 community nursing services	**Qn:** Log-in to the system data, questionnaire. **Ql:** In-depth interview.	Statistically and in descriptive format.	**Organizational aspect:** Efficiency and quality of care.	Evaluation
[51]	No	4 weeks	31	**Qn:** Survey with standardized questionnaires (Eheals, gad-7, OASIS, ASI-3, PDSS-SR, QLES-Q-SF, Nijmegen questionnaire).	Statistically and in descriptive format.	**Organizational aspect:** Feasibility and efficacy.	Pilot study
[52]	No	6-months	46	**Qn:** Survey (BDI-II and others).	Statistically	**Clinical aspects:** Depressive symptom severity, medication adherence.	Pilot study
[53]	No	12 weeks	128	**Qn:** Survey with standardized questionnaire (HRSD-24, SF-12, BADS-SF QIDS-CR, HADS-A, WHO-5).	Statistically	**Clinical aspect:** Depressive symptom severity, anxiety. **Human and social aspect:** QoL.	Pilot study
[54]	No	12 months	43	**Qn:** Survey with standardized questionnaire (FIM).	Statistically	**Human and social aspect:** Independence.	Pilot study
[55]	No	8 weeks	8	**Qn:** Survey with standardized questionnaire (QIDS-C,	Statistically	**Clinical aspect:** Depression symptom severity, anxiety. **Human and social aspect:** Satisfaction,	Pilot study

				PHQ-9, GAD-7).<br>**Ql:** Telephone interview.		**Technological aspect:** Technical feasibility, functional reliability.	
[56]	No	Not mentioned	5 NHS acute hospital and mental health trusts.	**Qn:** Survey.<br>**Ql:** Semi-structured interviews, documents, field notes, observation.	Descriptive interpretation of data.	**Organizational aspect:** Implementation and adoption of electronic health records.	Evaluation
[57]	Relevance - Appropriateness - Transparency - Soundness (RATS)	10 months	Preoperative clinics in 14 territorial health-boards of Scotland	**Ql:** Interviews, workshops.	Descriptive interpretation of data.	**Organizational aspect:** Implementation of an integrated preoperative care pathway and regional electronic clinical portal.	Evaluation
[58]	No	6 weeks	86 in Study-I and 39 in Study-II	**Qn:** Likert scale questionnaire).<br>**Ql:** Semi-structured interview.	Statistically and in descriptive format (this paper includes the descriptive part only).	**Technological aspect:** System usefulness (expected and perceived), impact of reminders, ease of use.	Pilot study
[59]	No	6 weeks	7	**Qn:** Survey with standardized questionnaire (MADRS-S, BDI-II, ISI, WSAS, PTQ, AS-18) and single question for perceived usefulness.	Statistically and in descriptive format.	**Clinical aspect:** Depressive symptoms.<br>**Human and social aspect**: Treatment adherence, perceived usefulness.	Pilot study
[60]	CONSORT	12 weeks	562	**Qn:** Survey with standardized questionnaire (PHQ-9, MOS, GAD-7, IPAQ, WHODAS-II).	Statistically	**Clinical aspect**: Depression symptom severity, anxiety, physical activity, adherence.	Pilot study
[61]	Framework by Campbell and colleagues 2000)	6 weeks	20	**Qn:** Survey with standardized questionnaire (BPRS) and Structured clinical interview (SCID).	Statistically and in descriptive format (this paper includes the descriptive part only).	**Clinical aspect:** Psychiatric rating scale, DSM disorder.<br>**Human and social aspect:** Perception of the system.	Design and pretesting
[62]	No	4 weeks	27	**Qn:** Survey with standardized questionnaire (BDI-2, QIDS-SR,	Statistically and in descriptive format.	**Technological aspect:** System use and acceptability.	Pilot study

				EQ-5D-5L, DAS-SF12).			
[63]	No	4 months	42	**Ql:** Semi-structured and written interview.	Statistically and in descriptive format.	**Human and social aspect:** Empowerment and assistance in decision making. **Technological aspect:** Acceptance and willingness to use the system.	Pilot study
[64]	No	8 weeks	108	**Qn:** Survey with standardized questionnaires (Positive and Negative Syndrome scale and Global Impression Scale). **Ql:** Structured interview.	Statistically and in descriptive format.	**Clinical aspect:** Adherence to medication.	Pilot study
[65]	No	3 months	40	**Qn:** Survey with standardized questionnaires (WHO-QOL-Brief, Dutch empowerment questionnaire, Pearlin mastery scale, social network questionnaire, client satisfaction questionnaire, SUS).	Statistically and in descriptive format.	**Clinical aspect:** Mastery. **Human and social aspect:** QoL, empowerment, social cohesion, satisfaction with care.	Pragmatic trial

Note. AS-18: Affective Scale-rating scale-18; ASI-3: Anxiety Sensitivity Index-3; BADS-SF: Behavioral Activation for Depression-short Form; BDI-II: Beck Depression Inventory-II; BPRS: Psychiatric Rating Scale; CES-D: Center for Epidemiologic Studies Depression; COPE-index: Careres of Older People in Europe index; DASS-21: Depression Anxiety Stress Scale-21; DSM: Diagnostic and Statistical Manual (for mental disorder); Eheals: eHealth Literacy Scale; EQ-5D-5L: EuroQol; FIM: Functional Independence Measure; GAD-7: Generalized Anxiety Disorder-7; GSE: General Self-efficacy; HADS: Hospital Anxiety and Depression Scale; HRSD-24: Hamilton Rating Scale for Depression-24; IMR: Illness Management and Recovery; IPAQ: International Physical Activity Questionnaires; ISI: Insomnia Severity Index; KPD-38: Klinisch Psychologisches Diagnosesystem-38; MADRS-S: Self-reported Montgomery-Åsberg Depression Rating Scale; MHSES: Mental Health Self-efficacy Scale; MOS: Medical Outcome Study; OASIS: Outcome and Assessment Information Set; PDSS-SR: Panic Disorder Severity Scale; PHQ-9: Patient Health Questionnaire-9; PSS: Perceived Stress Scale; PTQ: Perseverative Thinking Questionnaire; QIDS-CR: Quick Inventory of Depressive Symptomatology; Ql: Qualitative; QLES-Q-SF: Quality of Life

Enjoyment and Satisfaction-short Form; QoL: Quality of Life; Qn: Quantitative; SANS: Assessment of Negative Symptoms; SCID: Structured Clinical Interview for Disorder; SF-12: Short Form-12; WHO-5: Wellbeing Index; WHODAS-II: World Health Organization Disability Assessment Schedule-II; WSAS: Work and Social Adjustment Scale.

References

20. Harrison V, Proudfoot J, Wee PP, Parker G, Pavlovic DH, Manicavasagar V. Mobile mental health: review of the emerging field and proof of concept study. J Ment Health 2011 Dec;20(6):509-524. PMID:21988230

42. Aschbrenner KA, Naslund JA, Gill LE, Bartels SJ, Ben-Zeev D. A qualitative study of client-clinician text exchanges in a mobile health intervention for individuals with psychotic disorders and substance use. J Dual Diagn 2016;12(1):63-71. PMID:26829356
43. Mohr DC, Duffecy J, Ho J, Kwasny M, Cai X, Burns MN, Begale M. A randomized controlled trial evaluating a manualized TeleCoaching protocol for improving adherence to a web-based intervention for the treatment of depression. PLoS One 2013 Aug;8(8):e70086. PMID:23990896
44. Kleiboer A, Donker T, Seekles W, van Straten A, Riper H, Cuijpers P. A randomized controlled trial on the role of support in Internet-based problem solving therapy for depression and anxiety. Behav Res Ther 2015 Sep;72:63-71. PMID:26188373
45. Cristancho-Lacroix V, Moulin F, Wrobel J, Batrancourt B, Plichart M, De Rotrou J, Cantegreil-Kallen I, Rigaud A. A web-based program for informal caregivers of persons with Alzheimer’s disease: an iterative user-centered design. JMIR Res Protoc 2014 Sep;3(3):e46. PMID:25263541
46. Klein B, Meyer D, Austin DW, Kyrios M. Anxiety online: a virtual clinic: preliminary outcomes following completion of five fully automated treatment programs for anxiety disorders and symptoms. J Med Internet Res 2011 Nov;13(4):e89. PMID:22057287
47. Pratt SI, Naslund JA, Wolfe RS, Santos M, Bartels SJ Automated telehealth for managing psychiatric instability in people with serious mental illness. J Ment Health 2015;24(5):261-5. PMID:24988132
48. Zimmer B, Moessner M, Wolf M, Minarik C, Kindermann S, Bauer S. Effectiveness of an Internet-based preparation for psychosomatic treatment: Results of a controlled observational study. J Psychosom Res 2015 Nov;79(5):399-403. PMID:26526315
49. Ali L, Krevers B, Sjöström N, Skärsäter I. Effectiveness of web-based versus folder support interventions for young informal carers of persons with mental illness: a randomized controlled trial. Patient Educ Couns 2014 Mar;94(3):362-71. PMID:24341963
50. Bergmo TS, Ersdal G, Rødseth E, Berntsen G. Electronic messaging to improve information exchange in primary care. Proceedings 5th International Conference on eHealth, Telemedicine, and Social Medicine eTELEMED; 2013 Feb 24–Mar 1; Nice, France. ISBN: 978-1-61208-252-3

51. Pham Q, Khatib Y, Stansfeld S, Fox S, Green T. Feasibility and efficacy of an mhealth game for managing anxiety: “flowy” randomized controlled pilot trial and design evaluation. Games Health J 2016 Feb;5(1):50-67. PMID:26536488

52. Meglic M, Furlan M, Kuzmanic M, Kozel D, Baraga D, Kuhar I, Kosir B, Iljaz R, Novak Sarotar B, Dernovsek MZ, Marusic A, Eysenbach G, Brodnik A. Feasibility of an eHealth service to support collaborative depression care: results of a pilot study. J Med Internet Res 2010 Dec;12(5):e63. PMID:21172765

53. Ebert DD, Lehr D, Baumeister H, BoB L, Riper H, Cuijpers P, Reins JA, Buntrock C, Berking M. GET.ON Mood enhancer: efficacy of Internet-based guided self-help compared to psychoeducation for depression: an investigator-blinded randomised controlled trial. Trials 2014 Jan;15:39. PMID:24476555

54. Skidmore ER, Butters M, Whyte E, Grattan E, Shen J, Terhorst L. Guided training relative to direct skill training for individuals with cognitive impairments after stroke: a pilot randomized trial. Arch Phys Med Rehabil 2017 Apr;98(4):673-680. PMID:27794487

55. Burns MN, Begale M, Duffecy J, Gergle D, Karr CJ, Giangrande E, Mohr DC. Harnessing context sensing to develop a mobile intervention for depression. J Med Internet Res 2011 Jul-Sep;13(3):e55. PMID:21840837

56. Robertson A, Cresswell K, Takian A, Petrakaki D, Crowe S, Cornford T, Barber N, Avery A, Fernando B, Jacklin A, Prescott R, Klecun E, Paton J, Lichtner V, Quinn C, Ali M, Morrison Z, Jani Y, Waring J, Marsden K, Sheikh A. Implementation and adoption of nationwide electronic health records in secondary care in England: qualitative analysis of interim results from a prospective national evaluation. Br Med J 2010 Sep;341:c4564. PMID:20813822

57. Bouamrane MM, Mair FS. Implementation of an integrated perioperative care pathway and regional electronic clinical portal for preoperative assessment. BMC Med Inform Decis Mak 2014 Nov;14:93. PMID:25407812

58. Langrial SU, Lappalainen P. Information systems for improving mental health: six emerging themes of research. Information Systems. Proceedings of the 20th Pacific Asia Conference on Informatics Systems (PACIS); 2016 Jun 27–Jul 1; Chiayi, Taiwan. ISBN: 9789860491029

59. Holländare F, Eriksson A, Lövgren L, Humble MB, Boersma K. Internet-based cognitive behavioral therapy for residual symptoms in bipolar disorder type II: a single-subject design pilot study. JMIR Res Protoc 2015 Apr;4(2):e44. PMID:25908235

60. Glozier N, Christensen H, Naismith S, Cockayne N, Donkin L, Neal B, Mackinnon A, Hickie I. Internet-delivered cognitive behavioural therapy for adults with mild to moderate depression and high cardiovascular disease risks: a randomised attention-controlled trial. PLoS One 2013;8(3):e59139. PMID:23555624

61. Lederman R, Wadley G, Gleeson J, Bendall S, Álvarez-Jiménez M. Moderated online social therapy: designing and evaluating technology for mental health. ACM Trans Comput Hum Interact (TOCHI) 2014 Feb;21(1):1-26. doi:10.1145/2513179

62. Burton C, Szentagotai Tatar A, McKinstry B, Matheson C, Matu S, Moldovan R, Macnab M, Farrow E, David D, Pagliari C, Serrano Blanco A, Wolters M, Help4Mood Consortium. Pilot randomised controlled trial of Help4Mood, an

embodied virtual agent-based system to support treatment of depression. J Telemed Telecare 2016 Sep;22(6):348-55. PMID:26453910

63. Schaller S, Marinova-Schmidt V, Gobin J, Criegee-Rieck M, Griebel L, Engel S, Stein V, Graessel E, Kolominsky-Rabas PL. Tailored e-Health services for the dementia care setting: a pilot study of ‘eHealthMonitor. BMC Med Inform Decis Mak 2015 Jul;15:58. PMID:26215731

64. Frangou S, Sachpazidis I, Stassinakis A, Sakas G. Telemonitoring of medication adherence in patients with schizophrenia. Telemed J E Health 2005 Dec;11(6):675-83. PMID:16430387

65. de Wit J, Dozeman E, Ruwaard J, Alblas J, Riper H. Web-based support for daily functioning of people with mild intellectual disabilities or chronic psychiatric disorders: A feasibility study in routine practice. Internet Interv 2015 May;2(2):161-168. doi: 10.1016/j.invent.2015.02.007
